# Novel associations between sex hormones and diabetic vascular complications in men and postmenopausal women: a cross-sectional study

**DOI:** 10.1186/s12933-019-0901-6

**Published:** 2019-07-31

**Authors:** Chiyu Wang, Wen Zhang, Yuying Wang, Heng Wan, Yi Chen, Fangzhen Xia, Kun Zhang, Ningjian Wang, Yingli Lu

**Affiliations:** Institute and Department of Endocrinology and Metabolism, Shanghai Ninth People’s Hospital, Shanghai Jiao Tong University School of Medicine, Shanghai, 200011 China

**Keywords:** Diabetic macrovascular complications, Diabetic kidney disease, Testosterone, Estradiol, Dehydroepiandrosterone

## Abstract

**Background:**

Associations between sex hormones and vascular remodeling have been extensively studied, but the results vary widely among different races and sex. We aimed to investigate whether total testosterone (TT), estrogen (E2), and dehydroepiandrosterone (DHEA) associate with macrovascular complications and diabetic kidney disease (DKD) among community-dwelling patients with diabetes.

**Methods:**

A total of 4720 participants with type 2 diabetes were recruited from Shanghai, China. Common carotid artery (CCA) plaques and diameter were assessed by ultrasound. Cardiovascular disease (CVD) was defined by prior diagnosis of coronary heart disease, myocardial infarction or stroke. DKD was defined according to the ADA Guidelines.

**Results:**

(1) In men, TT was negatively associated with CCA diameter (regression coefficient (β) − 0.044, 95% CI − 0.087, 0). E2 levels were positively associated with CVD and CCA plaque prevalence (OR 1.151, 95% CI 1.038, 1.277 and OR 1.13, 95% CI 1.017, 1.255, respectively). DHEA was negatively associated with CVD (OR 0.809, 95% CI 0.734, 0.893). In postmenopausal women, TT levels were negatively associated with CCA diameter (β − 0.046, 95% CI − 0.083, − 0.010) and positively associated with CVD (OR 1.154, 95% CI 1.038, 1.284). (2) In both men and postmenopausal women, TT levels were negatively associated with the albumin/creatinine ratio and DKD (β − 0.098, 95% CI − 0.154, − 0.043 and OR 0.887, 95% CI 0.790, 0.997 vs. β − 0.084, 95% CI − 0.137, − 0.031 and OR 0.822, 95% CI 0.731, 0.924, respectively) and DHEA levels were positively associated with DKD (OR 1.167, 95% CI 1.038, 1.313 vs. OR 1.251, 95% CI 1.104, 1.418, respectively).

**Conclusions:**

Our study indicates that macrovascular complications were associated with low TT, DHEA and high E2 in men and with high TT in postmenopausal women. DKD was associated with low TT and high DHEA levels in both genders. Sex hormone replacement therapy requires careful and comprehensive consideration.

*Trial registration* ChiCTR1800017573, http://www.chictr.org.cn. Registered 04 August 2018

**Electronic supplementary material:**

The online version of this article (10.1186/s12933-019-0901-6) contains supplementary material, which is available to authorized users.

## Background

There were an estimated 425 million cases of adult diabetes worldwide in 2017 and more than 30% of these cases occurred in China [1]. Diabetes is a disorder of glucose metabolism that affects multiple organ systems. Sustained hyperglycemia can cause extensive vascular damage to the cardiovascular system, retina, kidneys, and nerves, and can cause various complications. Vascular lesions are important complications of chronic diabetes and are the leading cause of death in patients with diabetes. Approximately 27.2% and 22.3% of patients with diabetes have macrovascular complications and renal disease, respectively [2]. Prevention and control of vascular complications is key in lifelong diabetes treatment.

Gender-specific research is critical in elucidating the etiology of diabetes and developing targeted prevention and treatment strategies. In addition to regulating the reproductive system and maintaining secondary sexual characteristics, endogenous sex hormones regulate metabolic homeostasis through binding to their nuclear receptors or through their nongenomic signaling pathways [3–5].

Generally, men have higher prevalence of CVD than women, although postmenopausal women have a higher incidence of CVD and cardiovascular mortality than age-matched men [6], reflecting sexual dimorphism. In men, a meta-analysis showed that low testosterone and high estradiol levels were risk factors for cardiovascular mortality [7]. Physiological DHEA concentrations acutely increase nitric oxide release from intact vascular endothelial cells, which might underlie some of the cardiovascular protective effects proposed for DHEA [8, 9]. In postmenopausal women, the association between endogenous testosterone and CVD risk has not been concluded.

There is no evidence of gender differences in the association of eGFR with urinary albumin; however, the incidence of renal replacement therapy in men exceeds that of women [10, 11]. With the progression of CKD, the prolactin clearance rate is reduced, causing hyperprolactinemia by affecting the normal cycle of hypothalamic gonadotropin releasing hormone (GnRH); therefore, the basal secretion of LH and FSH appears to remain normal, but the pulse release rhythm is impaired, which in turn affects sex hormone levels. 17β-estradiol can be used to treat glomerular sclerosis, tubulointerstitial fibrosis and albuminuria in rats with streptozocin (STZ)-induced diabetes [12–14], but the opposite conclusion was observed in Cohen rats, in which oophorectomy reduced diabetic nephropathy and 17β-estradiol aggravated diabetic nephropathy [15]. These studies indicate that the role of sex hormones in DKD remains controversial and requires further research.

In summary, the association of sex hormones and macrovascular complications is still controversial. Nevertheless, studies of sex hormones and DKD are very rare and promising. This study is based on community-dwelling patients with diabetes and aims to determine the association of sex hormones and vascular complications in different genders and systems.

## Methods

### Study population

**Fig. 1:**
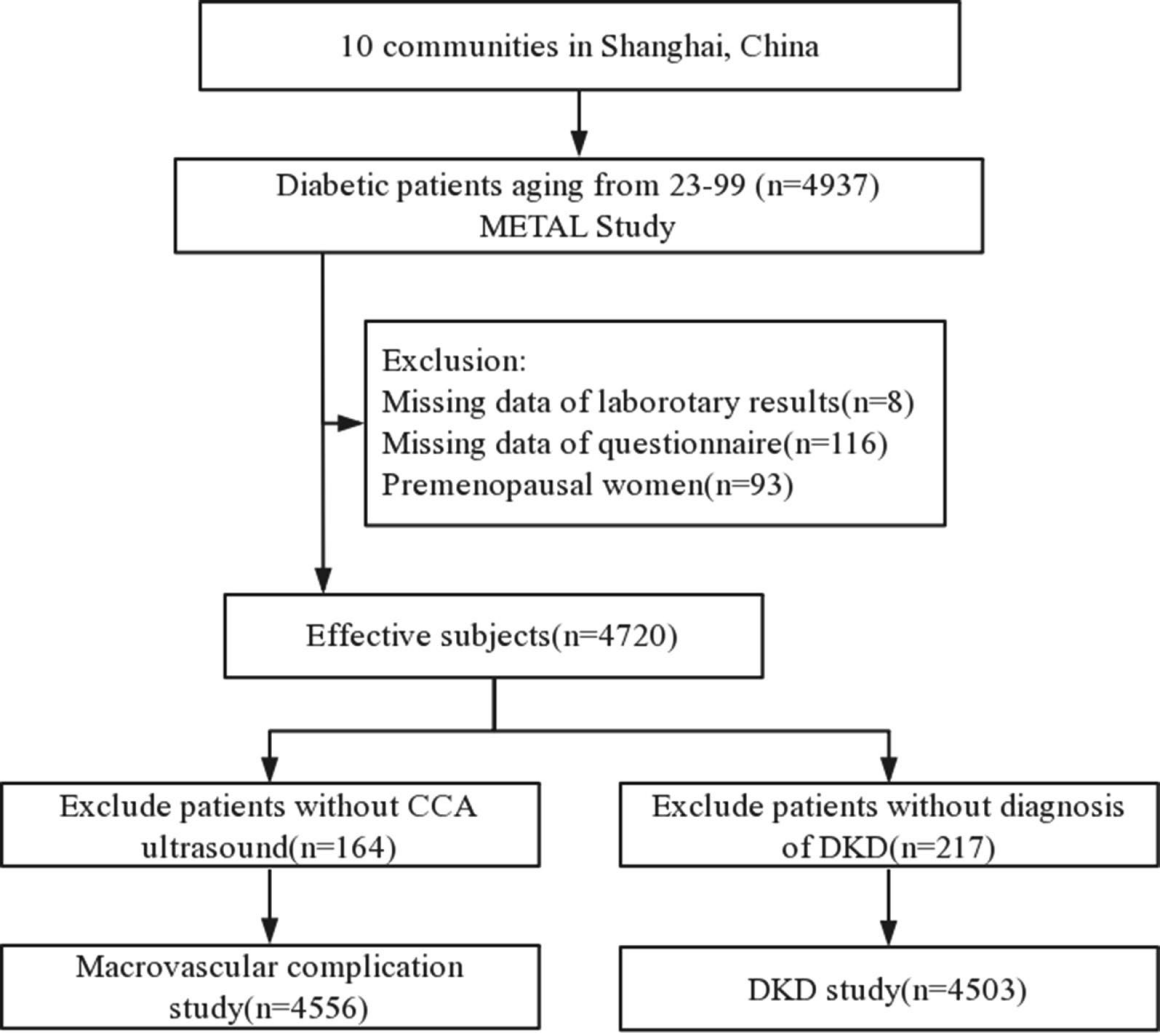
Flowchart of the inclusion and exclusion of participants

The cross-sectional METAL study (Environmental Pollutant Exposure and Metabolic Diseases in Shanghai, http://www.chictr.org.cn, ChiCTR1800017573) was performed from May to August, 2018. Participants were enrolled from ten communities in Shanghai, China. Chinese citizens aged 18 years and older who had lived at their current residence for ≥ 6 months were invited to participate in our study. A total of 4937 people with type 2 diabetes 23–99 years of age were included in our investigation. We excluded participants who were missing laboratory results (n = 8) or questionnaires (n = 116) and 93 premenopausal women. A total of 4720 participants were enrolled in the METAL study. For the macrovascular complications study, another 164 participants were excluded due to missing carotid artery ultrasound measurement or CVD diagnosis data. Similarly, 217 participants were excluded due to missing DKD diagnosis (Fig. 1). A woman was considered postmenopausal if she confirmed menopause on the questionnaire or was ≥ 60 years old or ≥ 55 years old with FSH ≥ 25 IU/L [16].

The study protocol was approved by the Ethics Committee of Shanghai Ninth People’s Hospital, Shanghai JiaoTong University School of Medicine. All procedures abided by the ethical standards of the responsible committee on human experimentation (institutional and national) and by the Helsinki Declaration of 1975, as revised in 2008. Informed consent was obtained from all participants for inclusion in the study.

### Measurements

A standard questionnaire was administered by the same well-trained and experienced personnel as in the SPECT-China study [17, 18] to obtain information on sociodemographic characteristics, personal and family medical history, and lifestyle risk factors. Clinical examinations, including weight, height and blood pressure, were conducted by a trained staff group according to a standard guideline. Serum samples were obtained between 6:00 a.m. and 9:00 a.m. after fasting for at least 8 h. Venipuncture was performed in the median cubital vein, and centrifugation and dispensing were completed within 1 h. Urine samples were obtained under normative retention guidelines in the morning on the day of epidemiological investigation, taken to the study site and registered by bar code. All samples were cold-chained, stored and transported to a central laboratory for testing within 2–4 h. The remaining samples were packaged and stored at − 20 °C.

Glycated hemoglobin (HbA1c) was assessed by high-performance liquid chromatography (MQ-2000PT, China). Plasma glucose and lipid profiles including total cholesterol (TC), triglycerides (TG), high-density lipoprotein (HDL) and low-density lipoprotein (LDL) were measured by Beckman Coulter AU 680 (Germany). Serum creatinine was measured by the picric acid method (Coulter AU 680). Insulin and DHEA were detected by chemiluminescence (Abbott i2000 SR, USA). TT, E2, FSH and LH were measured by electrochemiluminescence (Roche E601, Switzerland). Urine creatinine was measured by the enzymic method and urine microalbumin was measured by immunoturbidimetry (AU 680). The minimal detectable limits for each hormone were as follows: 0.087 nmol/L (total T), 18.4 pmol/L (E2), 3.0 pg/mL (DHEA) and 0.1 IU/L (FSH and LH). The inter-assay coefficients of variation were 8.33% (TT, E2, FSH, and LH) and 4.21% (DHEA). The intra-assay coefficients of variation were 6.25% (TT, E2, FSH, and LH) and 1.98% (DHEA).

The carotid artery ultrasound was assessed by same batch of trained sonographers who were authenticated by the Ministry of Health of China and blinded to any clinical conditions of the participants. All of sonographers received formal training, including performing carotid ultrasound on the same patients before the study began to achieve inter-observer coefficient of variation less than 10%. CIMT was measured by high-resolution B-mode ultrasound imaging (M7, Mindray ShenZhen, P.R. China) with a linear 10 MHz transducer. The measurement method was based on the consensus statement from the American Society of Echocardiography Carotid Intima-Media Thickness Task Force [19]. Manipulating the transducer maximized the lumen diameter in the longitudinal plane. The scan range included the bilateral common carotid artery, internal carotid artery and bifurcation. CIMT was measured during end diastole on the far wall and the mean value of bilateral CIMT was used for analysis. All the CCA diameter data were interadventitial diameter, measured between the leading edge of the adventitia- media echo of the near wall and the leading edge of the media-adventitia echo of the far wall, based on an average of seven end-diastolic diameter measurements at 5–10 mm proximal to the carotid bulb.

## Definition

Diabetes was defined as FPG ≥ 7.0 mmol/L, HbA1c ≥ 6.5% or diagnosis by a healthcare professional [20]. Dyslipidemia was defined as TC ≥ 6.22 mmol/L, TG ≥ 2.26 mmol/L, LDL-C ≥ 4.14 mmol/L, HDL-C < 1.04 mmol/L, or self-reported previous diagnosis of hyperlipidemia by a physician [21]. Hypertension was defined by systolic blood pressure ≥ 140 mmHg, diastolic blood pressure ≥ 90 mmHg, or self-reported previous diagnosis of hypertension by a physician. Body mass index (BMI) was calculated as the weight in kilograms divided by height in meters squared. Current smoking was defined as having smoked at least 100 cigarettes in a person’s lifetime and currently smoking cigarettes [22].

The CVD outcome was defined as a self-reported diagnosis including coronary heart disease, myocardial infarction or stroke. Present CCA plaque was identified as focal thickening (≥ 1.5 mm) of the artery wall [23]. A high urine albumin creatinine ratio (ACR) was defined as ACR > 30 mg/g. The estimated glomerular filtration rate (eGFR) was calculated according to the Chronic Kidney Disease Epidemiology Collaboration Research (CKD-EPI) equation for “Asian origin” [24], and DKD was defined as ACR > 30 mg/g or eGFR < 60 mL/min/1.73 m^2^ [25].

## Statistical analysis

The database was collected by EpiData 3.0 and analyzed using SPSS Statistics V.22 (IBM Corporation, Armonk, NY, USA). A P value < 0.05 was considered significant (two sided). Continuous variables were expressed as the mean ± SD or median (interquartile range) and categorical variables as percentages (%). Continuous variables were compared using Student’s t-test.

Sex hormone levels were equally divided into quartiles, with the first quartile (Q1) representing the lowest quartile and the fourth quartile (Q4) being the highest. Since serum E2 in postmenopausal women was relatively low with left-skewed distribution, the logarithmic transformation of E2 (lnE2) was used in this study. A 1 SD increment was used to reflect continuous changes of sex hormones. Linear (continuous dependent variables) and logistic (categorical dependent variables) regression were used, and the results were displayed as a regression coefficient (β) (95% CI) or odds ratio (OR) (95% CI), respectively.

For the association between sex hormones and macrovascular complications, the model was adjusted for age, duration of diabetes, current smoking, BMI, HbA1c, dyslipidemia, hypertension, antihypertensive medication, statin use or antiplatelet therapy, FSH, LH and other sex hormones. For the association between sex hormones and DKD, the model was adjusted for age, duration of diabetes, current smoking, BMI, HbA1c, dyslipidemia, hypertension, eGFR, FSH, LH and other sex hormones.

## Results

The general characteristics were assessed among respondents with diabetes (2223 men and 2497 postmenopausal women) in the METAL study. In men, the average age was 69.6 ± 8.12 years, the average duration of diabetes was 11.38 ± 8.13 years, the percentage of smokers was 36.3%, the prevalence of CVD was 35.7%, and the prevalence of DKD was 27.9%. In postmenopausal women, the average age was 69.5 ± 7.63 years, the average duration of diabetes was 11.86 ± 8.58 years, the percentage of smokers was 2.4%, the prevalence of CVD was 39.5%, and the prevalence of DKD was 28.5%.

### Sex-specific characteristics of participants by CVD condition in a macrovascular complications study

The clinical characteristics of patients with diabetes grouped according to gender and CVD conditions are summarized in Table 1. Patients with a history of CVD were older, had a longer period of diabetes and a higher BMI index, increased prevalence of hypertension, dyslipidemia and CCA plaques than those without CVD. The differences were statistically significant in both men and postmenopausal women (all P < 0.05). Sex hormone levels in patients with/without CVD history exhibited gender differences. In men, subjects with a history of CVD had higher E2, FSH, and LH levels and lower DHEA levels. In postmenopausal women, those with a history of CVD had higher TT levels and lower DHEA levels (all P < 0.05).

**Table 1:** General characteristics among participants with and without CVD

	Men		Postmenopausal women	
	Without CVD	With CVD	Without CVD	With CVD
N	1375	772	1455	954
Demographic parameter				
Age, years	65.99 ± 8.77	70.19 ± 7.94*	66.24 ± 7.43	69.78 ± 7.61*
Duration of diabetes, years	9.6 ± 7.68	11.2 ± 8.05*	9.22 ± 7.64	11.66 ± 8.52*
HbA1c, %	7.56 ± 1.41	7.62 ± 1.41	7.38 ± 1.37	7.45 ± 1.3
BMI, kg/m^2^	24.84 ± 3.41	25.28 ± 3.14*	24.61 ± 3.82	25.28 ± 3.72*
Smoking, %	39.7	29.3*	2.6	2.2
Hypertension, %	73.5	86.3*	75.5	87.2*
Dyslipidemia, %	59.9	70.5*	57.6	65.8*
Sex hormone level				
TT, nmol/L	15 ± 5.86	14.5 ± 6	0.59 ± 0.43	0.65 ± 0.64*
E2, pmol/L	114 (89.62, 138.9)	121.15 (95.47, 144.4)*	29.38 (9.18, 50.15)	31.67 (9.18, 53.21)
DHEA, pg/mL	202.39 ± 103.71	167.88 ± 89.1*	132.98 ± 67.91	124.76 ± 67.77*
FSH, IU/L	12 ± 9.94	13.53 ± 12.42*	55.28 ± 23.02	55.84 ± 24.35
LH, IU/L	8.27 ± 5.03	8.99 ± 6.43*	26.05 ± 11.42	26.38 ± 12.62
Carotid artery ultrasound				
CCA plaque, %	63.5	77.3*	42	52.7*
CCA diameter, mm	7.77 ± 0.88	7.92 ± 0.88*	7.48 ± 0.79	7.68 ± 0.78*
cIMT, mm	0.87 ± 0.15	0.89 ± 0.15*	0.81 ± 0.13	0.84 ± 0.14*

Normally distributed variables are expressed as mean ± standard deviation, non-normal variables are expressed as median (P25, P75) and categorical variables are expressed as percentage (%). The Student’s t test was used to compare the continuous variables of the normal distribution, the Mann–Whitney U test was used for the comparison of the non-normal distribution continuous variables, and the Pearson χ^2^ test was used for the comparison between the categorical variables

* P < 0.05, compared with those without CVD

### Association of sex hormone levels with vascular measurement and CVD

**Fig. 2:**
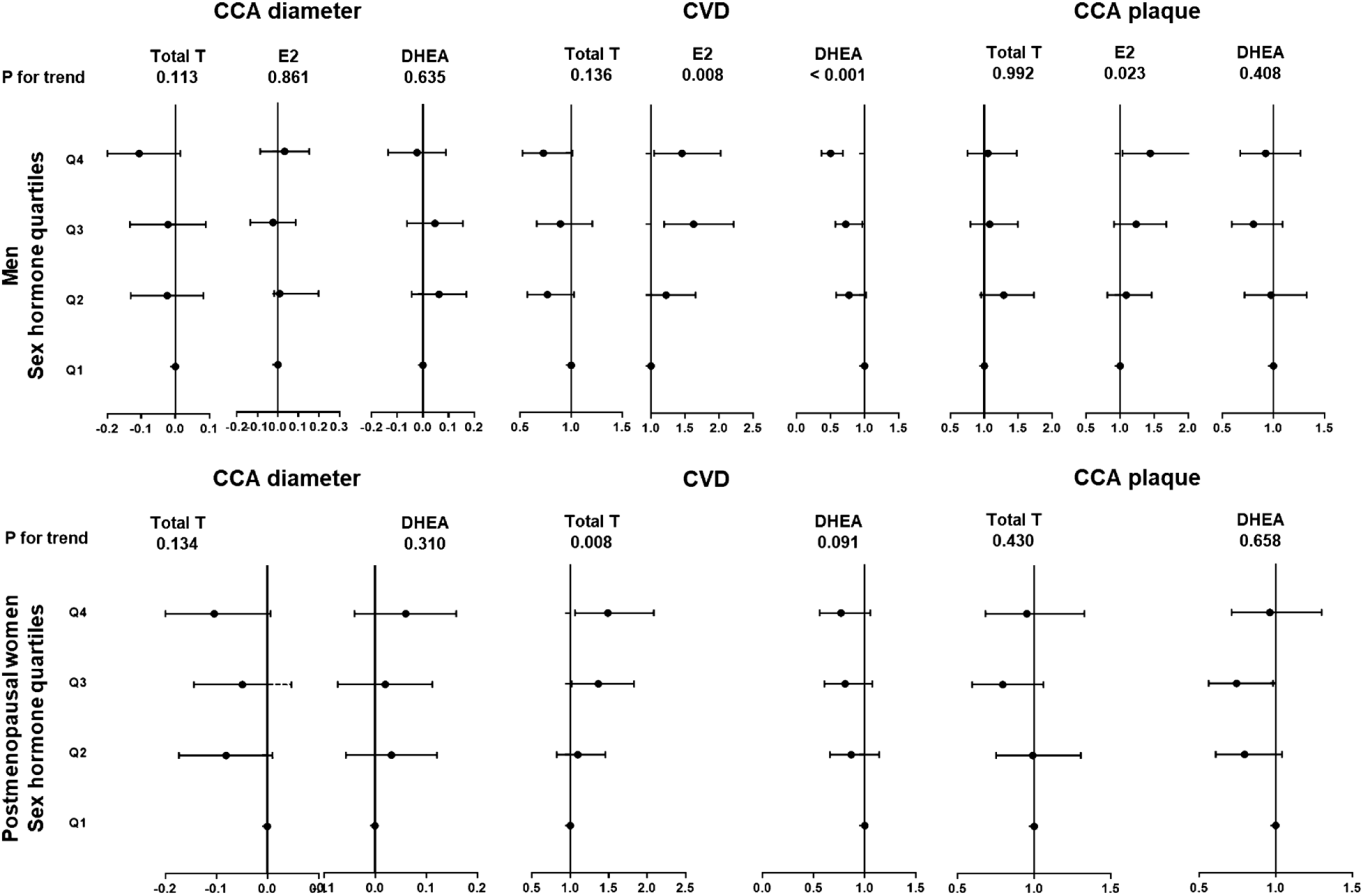
Association of sex hormone level quartiles with diabetic macrovascular complications in men and postmenopausal women. Linear (CCA diameter) and binary logistic (CVD and CCA plaque) regression analyses were used

Figure 2 summarizes the association of sex hormone levels and macrovascular complications in patients with diabetes. In men, TT was negatively associated with CCA diameter (regression coefficient (β) − 0.044, 95% CI − 0.087, 0, P 0.048). The OR (95% CI) of having CVD in binary logistic regression associated with E2 was 1.223 (0.903, 1.656) for the second quartile, 1.627 (1.195, 2.215) for the third quartile and 1.456 (1.046, 2.027) for the fourth quartile, compared with first quartile (P for trend 0.008). The OR (95% CI) of having CCA plaque associated with E2 was 1.09 (0.812, 1.463) for the second quartile, 1.237 (0.912, 1.678) for the third quartile and 1.443 (1.033, 2.015) for the fourth quartile, compared with first quartile (P for trend 0.023). Moreover, the OR (95% CI) of having CVD associated with DHEA was 0.773 (0.583, 1.024) for the second quartile, 0.723 (0.540, 0.967) for the third quartile and 0.501 (0.367, 0.684) for the fourth quartile, compared with first quartile (P for trend < 0.001). A 1 SD increment of DHEA was also strongly associated with CVD (OR 0.753, 95% CI 0.678, 0.859 P < 0.001).

In postmenopausal women, the OR (95% CI) of having CVD associated with TT was 1.097 (0.825, 1.457) for the second quartile, 1.365 (1.019, 1.830) for the third quartile and 1.49 (1.064, 2.086) for the fourth quartile, compared with first quartile (P for trend 0.008).

### Clinical characteristics of participants by gender and DKD condition in the DKD study

As shown in Table 2, compared with patients without DKD, both male and postmenopausal female patients with DKD were older and had longer duration of diabetes, higher HbA1c, BMI, ACR, hypertension and dyslipidemia morbidity and lower eGFR (all P < 0.05). For sex hormone levels, male participants with DKD had higher levels of TT, FSH, and LH (all P < 0.05). Postmenopausal female participants with DKD had higher levels of FSH and LH, and lower E2 (all P < 0.05).

**Table 2:** General characteristics among participants with and without DKD

	Men		Postmenopausal women	
	Without DKD	With DKD	Without DKD	With DKD
N	1528	592	1705	678
Demographic parameter				
Age, years	66.75 ± 8.26	69.47 ± 9.34*	66.94 ± 7.23	69.61 ± 8.54*
Duration of diabetes, years	9.36 ± 7.41	12.35 ± 8.47*	9.51 ± 7.88	11.94 ± 8.37*
HbA1c, %	7.41 ± 1.27	8.02 ± 1.6*	7.29 ± 1.26	7.71 ± 1.46*
BMI, kg/m^2^	24.77 ± 3.24	25.54 ± 3.41*	24.54 ± 3.71	25.75 ± 3.89*
Smoking, %	34.9	38.7	2.5	2.4
Hypertension, %	73.7	89.9*	76.1	90.7*
Dyslipidemia, %	61.5	68.4*	59	64.5*
Sex hormone levels				
TT, nmol/L	15.13 ± 5.89	13.99 ± 5.89*	0.61 ± 0.55	0.63 ± 0.46
E2, pmol/L	122.3 (173.9, 243.63)	110.98 (172.65, 242.88)	79.35 (117.7, 163.85)	77.78 (121.6, 171.4)*
DHEA, pg/mL	190.73 ± 96.65	192.03 ± 109.8	128.5 ± 66.62	131.72 ± 71.56
FSH, IU/L	11.77 ± 9.33	14.55 ± 13.97*	54.35 ± 22.12	58.39 ± 26.78*
LH, IU/L	7.87 ± 4.4	10.24 ± 7.6*	25.49 ± 10.63	27.99 ± 14.53*
Renal function index				
lnACR	2.19 ± 0.63	4.46 ± 1.26*	2.32 ± 0.64	4.27 ± 1.12*
High ACR, %	0	92.6*	0	91.8*
eGFR, mL/min/1.73 m^2^	93.51 ± 12.65	81.84 ± 23.75*	94.71 ± 12.15	84.98 ± 22.72*

Normally distributed variables are expressed as mean ± standard deviation, non-normal variables are expressed as median (P25, P75) and categorical variables are expressed as percentage (%). The Student’s t test was used to compare the continuous variables of the normal distribution, the Mann–Whitney U test was used for the comparison of the non-normal distribution continuous variables, and the Pearson χ^2^ test was used for the comparison between the categorical variables

* P < 0.05, compared with those without DKD

### Association of sex hormone levels with DKD

**Fig. 3:**
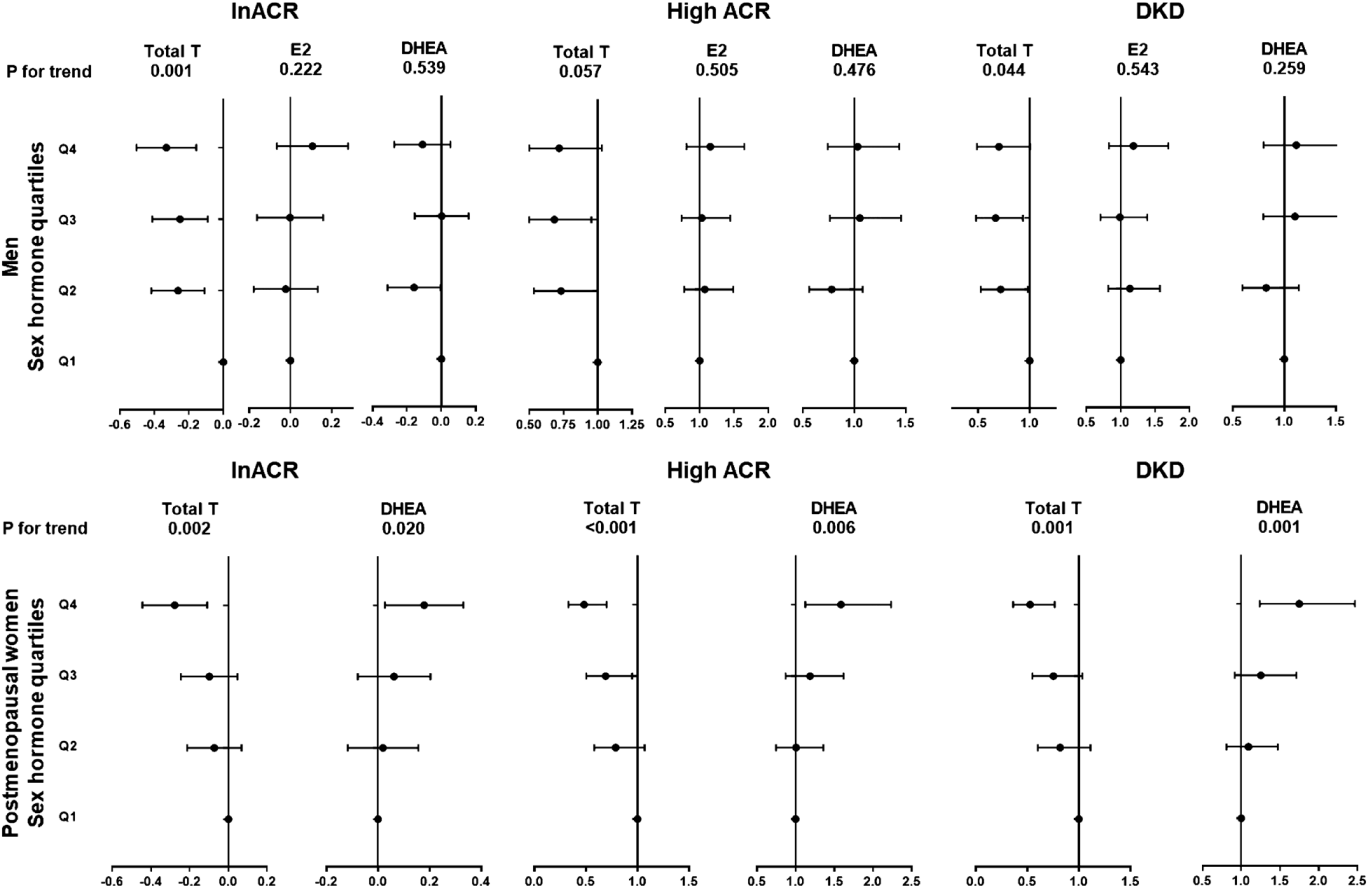
Association of sex hormone level quartiles with DKD in men and postmenopausal women. Linear (lnACR) and binary logistic (HighACR and DKD) regression analyses were used

The regression analysis results were summarized in Fig. 3.

In men, compared with first quartile, TT in the fourth quartile had the lowest β for lnACR (β − 0.331, 95% CI − 0.505, − 0.157) and OR for DKD (OR 0.702, 95% CI 0.489, 1.007). A 1 SD increment of TT was also negatively associated with lnACR, high ACR, and DKD (β − 0.117, 95% CI − 0.18, − 0.054, P < 0.001; OR 0.849, 95% CI 0.74, 0.974, P 0.02; OR 0.839, 95% CI 0.732, 0.962, P 0.012, respectively). A 1 SD increment of DHEA was positively associated with DKD (OR 1.167, 95% CI 1.038, 1.313, P 0.01).

In postmenopausal women, compared with first quartile, TT in the fourth quartile had the lowest β for lnACR (β − 0.278, 95% CI − 0.445, − 0.11) and OR for HighACR and DKD (OR 0.482, 95% CI 0.331, 0.701, OR 0.528, 95% CI 0.364, 0.766, respectively). A 1 SD increment of TT was also negatively associated with lnACR, HighACR and DKD (β − 0.075, 95% CI − 0.131, − 0.019, P 0.009; OR 0.773, 95% CI 0.655, 0.912, P 0.002; OR 0.787, 95% CI 0.668, 0.928, P 0.004, respectively). Compared with the first quartile, DHEA in the fourth quartile had the highest β for lnACR (β 0.179, 95% CI 0.027, 0.331) and OR for HighACR and DKD (OR 1.586, 95% CI 1.126, 2.234, OR 1.748, 95% CI 1.24, 2.465, respectively). Meanwhile, a 1 SD increment of DHEA was also positively associated with lnACR, HighACR and DKD (β 0.094, 95% CI 0.039,0.149, P 0.001; OR 1.231, 95% CI 1.086, 1.396, P 0.001; OR 1.251, 95% CI 1.104, 1.418, P < 0.001, respectively).

## Discussion

In this study, we evaluated the association between serum sex hormone levels and diabetic macro/microvascular complications in more than 4500 community-dwelling adults with diabetes in China. The results suggest that male patients with diabetes and higher serum E2 or lower DHEA and postmenopausal female patients with diabetes with higher serum TT have higher prevalence of macrovascular complications, while both male and postmenopausal female patients with lower serum TT or higher DHEA levels have higher prevalence of DKD. To the best of our knowledge, our study is the first to evaluate the prevalence of diabetic complication and emphasize the relationship between sex hormone levels and diabetic vascular complications in different genders.

Clinical manifestations indicate sex-specific differences in diabetic complications. Women with diabetes bear greater relative risk of CHD (1.40) and stroke (1.27) compared with men for both fatal and nonfatal events. Furthermore, men with diabetes suffer faster progression of DKD and more dialysis therapy/kidney transplantations than women, while women with diabetes have a higher risk of proteinuria and renal disease than men [26].

### Sex hormones and diabetic macrovascular complications

Due to the combined effects of insulin resistance, hyperglycemia and related metabolic abnormalities, patients with type 2 diabetes are particularly susceptible to atherosclerotic cardiovascular and cerebrovascular diseases. Identifying and detecting atherosclerosis early in the subclinical phase can help identify strategies to prevent disease progression.

### Testosterone and CVD

A large cohort study of men without known CVD indicated that TT levels are associated with aortic rigidity and that low testosterone concentrations have a more pronounced effect on aortic stiffness in younger and higher blood pressure subjects [27]. Testosterone regulates the NO-cGMP signaling pathway, and basic studies have shown that expression of NO synthase (NOS2) is increased and the cGMP-specific phosphodiesterases (PDE)5, PDE6 and PDE9 are upregulated after continuous use of testosterone for 2 weeks [28]. These results identify low testosterone as a marker of arterial injury in men. Interestingly, the association of TT levels and macrovascular complications in men with diabetes was not remarkable according to our study. On the other hand, the relationship between endogenous testosterone, CVD risk and all-cause mortality has not been established, especially in older women [29]. Clinically, the results of studies on the relationship between sex hormone levels and cardiovascular disease in postmenopausal women vary widely. High [30, 31] and low [32, 33] androgen levels are associated with an increased risk of cardiovascular disease. A recent study proposed that in women with PCOS, baseline testosterone levels did not predict CVD risk [34]. A prospective study suggest that higher levels of endogenous testosterone may mediate increased risk of cardiovascular disease in postmenopausal women [35, 36]. Our study indicated that TT levels were positively associated with CVD prevalence. Among CVD risks, CHD was more strongly associated with hormones than stroke (Additional file 1: Table S1).

### Estrogen and CVD

In some animal trials, estrogen is thought to regulate blood lipids, inhibit blood vessel response to damage, increase vasodilation, regulate blood clotting, antagonize lipid peroxidation, and maintain body weight and glucose homeostasis [37, 38], and among postmenopausal women, estrogens have a stabilizing and protective effect on the carotid arteries, as demonstrated by fewer inflammatory cells within the plaque cap of females compared to males [39]. However, limited epidemiological investigations have shown that higher E2 levels are associated with increased risk of CVD and CV mortality in men [7]. The protective effect of estrogen over the preceding 50 years could be considered a factor that explains the sex difference in myocardial function in the middle aged population [40]. In fact, the interaction of E2 and vascular disease exhibits puzzling inconsistency, which is the so called the “estrogen paradox” [41, 42]. The relationship of estradiol and testosterone as a whole with cardiovascular disease is quite interesting and intriguing. A 12 years’ follow-up study of multi-ethnic postmenopausal female participants free of CVD at the baseline in the United States showed that a higher testosterone/estradiol ratio was associated with an elevated risk for incident CVD, CHD, and HF events [43]. In our study, we found testosterone/estradiol ratio was negatively associated with CHD and stroke in men but has no significant association in postmenopausal women (Additional file 1: Table S1), associations between E2 and macrovascular complications in postmenopausal women with definite E2 value were not significant (Additional file 1: Table S2). Nevertheless, our study fills the gap between E2 and CVD at a cross-sectional level and gives a credible view for sex and gender differences in diabetic vascular complications, which needs to be further explored.

### DHEA and CVD

Many studies suggest that DHEA is beneficial for cardiovascular function. Epidemiological studies have shown that decreased levels of serum DHEA and DHEA-S are associated with increased cardiovascular risk [44–46]. Decreased circulating DHEA-S levels are associated with a decrease in activated protein C, which is an anticoagulant factor produced by the thrombin-thrombomodulin complex on vascular endothelial cells. The imbalance of the coagulation-fibrinolytic system is an important risk factor for vascular events, such as myocardial infarction [47]. Low serum DHEA predicts all-cause death, CVD and ischemic heart disease in older men. A randomized, double-blind trial found that DHEA replacement therapy in older men improved the arterial stiffness index, which became an independent risk factor for CVD with age. In the future, DHEA replacement therapy may partially reverse arterial aging and reduce the risk of CVD [48]. The mentioned follow up study suggested that DHEA was not associated to CVD [43]. Our data support this research, though the inclusion criteria, ethnicity of the participating population, duration of research, methods of measuring and adjusted model were different. DHEA was an important precursor of estrogen and androgen synthesis, the conversion of DHEA to sex hormones regulated by aromatase, SHBG and a series of signal pathways, both of our studies enrich the research field in different perspectives.

### Sex hormones and DKD

Decreased renal function is often positively associated with decreased testosterone levels. A cross-sectional study in Japan found that a decrease in testosterone levels was independently and negatively associated with eGFR decline [49]. Another study of male patients with type 2 diabetes and chronic kidney disease (CKD) found that 2/3 of patients with T2DM and CKD had free testosterone below the normal value, and another 10% of patients have a compensatory response to hypogonadism, which is likely to become high gonadotropin-type hypogonadism in the future [50]. Approximately 90% of male patients with stage 4 and 5 CKD have hypogonadism and/or compensatory hypogonadism [51]. Multiple studies have shown that patients with type 2 diabetes and CKD have lower TT concentrations compared with men without diabetes and CKD [52–54]. CKD in women progresses more slowly than in men [55, 56], but the relationship between sex, sex hormones and DKD is still unclear [57, 58]. With the progression of female CKD, menstrual cycle and fertility disorders become increasingly common. Once ESRD is reached, amenorrhea and infertility become the norm. ESKD women may undergo menopause at an earlier age [59]. The presence of CKD itself also seems to impair the production and function of sex hormones.

Our study found that the incidence of DKD was negatively associated with serum TT and positively associated with serum DHEA both in men and postmenopausal women with diabetes, which indicated endogenous testosterone and DHEA may play different roles in the macrovascular and microvascular systems. Sex hormones have different effects in increasing oxidative stress, activating the RAS system, and aggravating damaged renal fibrosis, but the related mechanisms still need to be further explored.

This study also has several limitations. First, the finding suggests that endogenous sex hormone levels associate with diabetic macrovascular complications and DKD. However, association does not indicate causation, and further cohort studies and follow-ups are needed. Second, all subjects in this study were enrolled from Shanghai, and most of the residents recruited were Han Chinese. Therefore, the results may not represent other regions in China or other ethnic groups. Third, the composition of carotid plaque by MRI helps to understand the progression of macrovascular atherosclerosis, but considering the sample size, in this study we use carotid artery ultrasound to evaluate the macrovascular condition.

## Conclusion

Associations between sex hormones and diabetic vascular complications are sexually dimorphic. Our study indicates that macrovascular complications were associated with low TT, DHEA, and high E2 in men and with high TT in postmenopausal women. DKD was associated with low TT levels in both genders. Furthermore, DHEA could be a risk factor for DKD but a protective factor for macrovascular complications, and sex hormone replacement therapy requires careful and comprehensive consideration. The relevant mechanism remains to be further studied.

## Additional file

**Additional file 1: Table S1.** Associations between TT, T/E2 and DHEA with CHD and stroke. **Table S2.** Associations between E2 and macrovascular complications in postmenopausal women with definite E2 value.

## Data Availability

The raw data supporting the conclusions of this manuscript will be made available by the authors, without undue reservation, to any qualified researcher.

## Abbreviations

ACR: urine albumin creatinine ratio
BMI: body mass index
CI: confidence interval
CIMT: carotid intima-media thickness
CKD: chronic kidney disease
CVD: cardiovascular disease
DHEA: dehydroepiandrosterone
DKD: diabetic kidney disease
E2: estradiol
eGFR: estimated glomerular filtration rate
FPG: fasting plasma glucose
FSH: follicle-stimulating hormone
HbA1c: hemoglobin A1c
HDL: high density lipoprotein
LDL: low density lipoprotein
LH: luteinizing hormone
OR: odds ratio
SCr: serum creatinine
SHBG: sex hormone binding globulin
TC: total cholesterol
TG: triglycerides
TT: total testosterone
T2DM: type 2 diabetes mellitus
