# Supplementary material for: Novel associations between sex hormones and diabetic vascular complications in men and postmenopausal women: a cross-sectional study
Source: Cardiovasc Diabetol. 2019 Jul 31;18:97. doi: 10.1186/s12933-019-0901-6 (PMC6668151; doi:10.1186/s12933-019-0901-6)
Supplement: Supplementary file 1 — Additional file 1: Table S1. Associations between TT, T/E2 and DHEA with CHD and stroke. Table S2. Associations between E2 and macrovascular complications in postmenopausal women with definite E2 value. [file 12933_2019_901_MOESM1_ESM.docx]

**Additional file**

**Table S1. Associations between TT, T/E2 and DHEA with CHD and stroke**

| **Men** | | | |
| --- | --- | --- | --- |
|  |  | coronary heart disease | stroke |
| Total testosterone, nmol/L | Q1(≤10.79) | Ref. | Ref. |
|  | Q2(10.80-14.08) | 0.982(0.711,1.357) | 0.73(0.52,1.025) |
|  | Q3(14.09-18.01) | 0.804(0.571,1.133) | 0.935(0.667,1.312) |
|  | Q4(≥18.02) | 0.738(0.509,1.069) | 0.825(0.571,1.193) |
|  | p for trend | 0.066 | 0.556 |
|  | per SD increase | 0.905(0.789,1.039) | 0.918(0.8,1.052) |
| Testosterone/Estradiol | Q1(≤0.093) | Ref. | Ref. |
|  | Q2(0.094-0.121) | 0.941(0.684,1.293) | 0.84(0.609,1.16) |
|  | Q3(0.122-0.158) | 0.743(0.529,1.043) | 0.667(0.472,0.941) |
|  | Q4(≥0.159) | 0.692(0.487,0.983) | 0.708(0.5,1.004) |
|  | p for trend | 0.019 | 0.025 |
|  | per SD increase | 0.831(0.711,0.971)* | 0.837(0.717,0.978)* |
| DHEA, pg/ml | Q1(≤119.69) | Ref. | Ref. |
|  | Q2(119.70-173.19) | 0.751(0.55,1.026) | 0.844(0.616,1.156) |
|  | Q3(173.20-241.89) | 0.637(0.456,0.889) | 0.782(0.562,1.088) |
|  | Q4(≥241.90) | 0.576(0.405,0.82) | 0.513(0.355,0.741) |
|  | p for trend | 0.001 | 0.001 |
|  | per SD increase | 0.79(0.688,0.907)* | 0.773(0.672,0.89)* |
| **Postmenopausal women** | | | |
|  |  | coronary heart disease | stroke |
| Total testosterone, nmol/L | Q1(≤0.29) | Ref. | Ref. |
|  | Q2(0.30-0.52) | 1.177(0.859,1.612) | 0.953(0.687,1.322) |
|  | Q3(0.53-0.81) | 1.397(1.012,1.928) | 1.088(0.779,1.52) |
|  | Q4(≥0.82) | 1.513(1.048,2.185) | 1.156(0.79,1.692) |
|  | p for trend | 0.017 | 0.34 |
|  | per SD increase | 1.067(0.952,1.197) | 1.094(0.964,1.241) |
| Testosterone/Estradiol | Q1(≤0.008) | Ref. | Ref. |
|  | Q2(0.009-0.017) | 1.297(0.959,1.753) | 1.297(0.959,1.753) |
|  | Q3(0.018-0.038) | 0.995(0.723,1.369) | 0.995(0.723,1.369) |
|  | Q4(≥0.039) | 1.307(0.955,1.788) | 1.307(0.955,1.788) |
|  | p for trend | 0.299 | 0.299 |
|  | per SD increase | 1.048(0.941,1.167) | 1.048(0.941,1.167) |
| DHEA, pg/ml | Q1(≤79.19) | Ref. | Ref. |
|  | Q2(79.20-118.94) | 0.994(0.743,1.33) | 0.943(0.696,1.277) |
|  | Q3(118.95-165.62) | 0.992(0.733,1.341) | 0.784(0.569,1.082) |
|  | Q4(≥165.63) | 0.764(0.547,1.068) | 0.863(0.611,1.219) |
|  | p for trend | 0.0155 | 0.246 |
|  | per SD increase | 0.899(0.796,1.015) | 0.96(0.847,1.088) |

*: p < 0.05. Data are presented as odds ratio (95% confidence interval).

The model was adjusted by age, duration of diabetes, current smoking, BMI, HbA1c, dyslipidemia, hypertension, antihypertensive medication, statin use or antiplatelet therapy, FSH, LH and other sex hormones.

**Table S2. Associations between E2 and macrovascular complications in postmenopausal women with definite E2 value**

| **Postmenopausal women** | | | | |
| --- | --- | --- | --- | --- |
|  |  | mean CCA diameter | CVD | plaque |
| E2, pmol/L | Q1(≤32.46) | Ref. | Ref. | Ref. |
|  | Q2(32.47-45.98) | -0.094(-0.203,0.015) | 1.088(0.775,1.528) | 0.868(0.624,1.208) |
|  | Q3(45.99-60.84) | -0.02(-0.128,0.088) | 1.206(0.863,1.686) | 0.737(0.529,1.025) |
|  | Q4(≥60.85) | -0.039(-0.153,0.076) | 1.152(0.806,1.648) | 0.801(0.563,1.138) |
|  | p for trend | 0.791 | 0.337 | 0.122 |
|  | per SD increase | -0.01(-0.051,0.031) | 1.039(0.914,1.181) | 0.885(0.779,1.004) |

N=1510, data are presented as odds ratio (95% confidence interval).

The model was adjusted by age, duration of diabetes, current smoking, BMI, HbA1c, dyslipidemia, hypertension, antihypertensive medication, statin use or antiplatelet therapy, FSH, LH and other sex hormones.
